# Integrin Signaling Pathways in Aortic Aneurysm and Dissection

**DOI:** 10.31083/RCM45868

**Published:** 2026-06-24

**Authors:** Ting Chen, Ping Li, Hongzhang Tong, Mengda Chen, Lingling Lu

**Affiliations:** ^1^Department of Radiology, The Affiliated People’s Hospital of Ningbo University, 315040 Ningbo, Zhejiang, China; ^2^Department of Pathology, The Affiliated People’s Hospital of Ningbo University, 315040 Ningbo, Zhejiang, China

**Keywords:** aortic aneurysm, aortic dissection, integrins, signal transduction, physiology

## Abstract

Aortic aneurysm (AA) and aortic dissection (AD) are life-threatening aortic diseases that primarily arise from medial degeneration. Ruptures are invariably associated with fatal outcomes; however, there are currently no clinically proven medications to prevent the progression of aortic aneurysm and dissection (AAD), and surgery remains the most effective approach to eliminating the high risk of aortic rupture. The dysfunction and depletion of vascular smooth muscle cells, along with inflammation and extracellular matrix (ECM) remodeling, are key mechanisms underlying AAD development. Integrins are heterodimeric transmembrane receptors that link the ECM to the cytoskeleton and transmit bidirectional cellular signals. Moreover, integrins maintain cell morphology and modulate numerous physiological and pathophysiological processes. Meanwhile, increasing evidence indicates that integrins play a vital role in AAD. This review outlines the basic biology of integrins, discusses the specific functions and mechanisms of integrins in AAD pathogenesis, and aims to identify reliable non-invasive biomarkers for acute AAD and potential targets to halt disease progression.

## 1. Introduction

Aortic aneurysm (AA) and aortic dissection (AD) are aortic diseases with high mortality rates [1]. The onset of aortic aneurysm and dissection (AAD) is driven by either genetic mutations (hereditary AAD), such as Marfan syndrome (MFS), or traditional risk factors such as hypertension, dyslipidemia, smoking, advanced age, and male sex (sporadic AAD) [2,3]. AA, a chronic and progressive focal dilation of the aorta that is usually asymptomatic, may culminate in AD or rupture and thus cause sudden death. Globally, AA accounted for 153,927 deaths in 2021 [4]. Anatomically, AA is classified into abdominal AA (AAA)—the most common form, typically of atherosclerotic origin—and thoracic AA (TAA), which is often genetically driven [5,6]. AD begins with an intimal tear that allows blood to flow into the aortic media, forming a false lumen [1]. The aortic adventitia may rupture and cause fatal hemorrhage at any time. AD may occur with or without an underlying AA and is most frequently observed in individuals aged 65–75 years, with an annual incidence of 35 cases per 100,000 individuals [7]. AD is classified, in the most widely used Stanford system, as type A if the ascending aorta is involved and type B if it is not [1]. Based on the symptom onset time, AD is divided into hyperacute (<24 h), acute (1–14 d), subacute (15–90 d), and chronic (>90 d) types. Type A acute AD has a high mortality rate; it increases by approximately 1% to 2% per hour, and about 50% of patients will die within the initial 48 h without surgical intervention [1]. To date, surgery remains the best approach to eliminate the high risk of aortic rupture, and no drugs have been proven clinically to halt AAD progression [1,8]. The development of AAD is closely associated with endothelial cell dysfunction, vascular smooth muscle cell (VSMC) depletion and phenotype switching, inflammatory cell infiltration, and extracellular matrix (ECM) remodeling [5,6,9,10,11,12]. Thus, dissecting the pathological mechanisms may provide effective strategies to treat AAD.

The ECM, a highly dynamic structure produced by resident cells, is an essential component that maintains the structural and functional integrity of the aortic wall. It is composed of polysaccharides such as hyaluronic acid, fibrous proteins (e.g., collagen, elastin, and fibronectin), glycoproteins, proteoglycans, and glycosaminoglycans. Its specific composition is determined by the diverse functions of the cell types within the aortic wall. In turn, the composition and integrity of the ECM regulate the function and survival of the cells residing within the aortic wall [13,14,15]. In disease states, profound ECM remodeling directly influences the fate of VSMCs, endothelial cells, and immune cells. These changes are the primary driving forces of AAD pathogenesis [11,16,17].

Integrins are heterodimeric transmembrane receptors that physically couple the ECM to the cytoskeleton, forming a mechanical bridge that allows cells to sense and respond to their physical surroundings. Beyond this structural function, they transmit bidirectional cellular signals across the membrane to affect cell behavior, including cell adhesion, proliferation, migration, and differentiation, in both physiological and pathological contexts [18,19]. Accumulating evidence implicates integrins in the initiation and progression of cardiovascular diseases, including AAD [20,21,22,23,24]. This review presents the basic biology of integrins and dissects their specific roles and mechanisms in the pathogenesis of AAD, aiming to identify reliable, non-invasive biomarkers for the diagnosis of acute AAD and find potential targets for preventing disease progression.

## 2. Overview of Integrins

### 2.1 Structure of Integrins

**Fig. 1.**
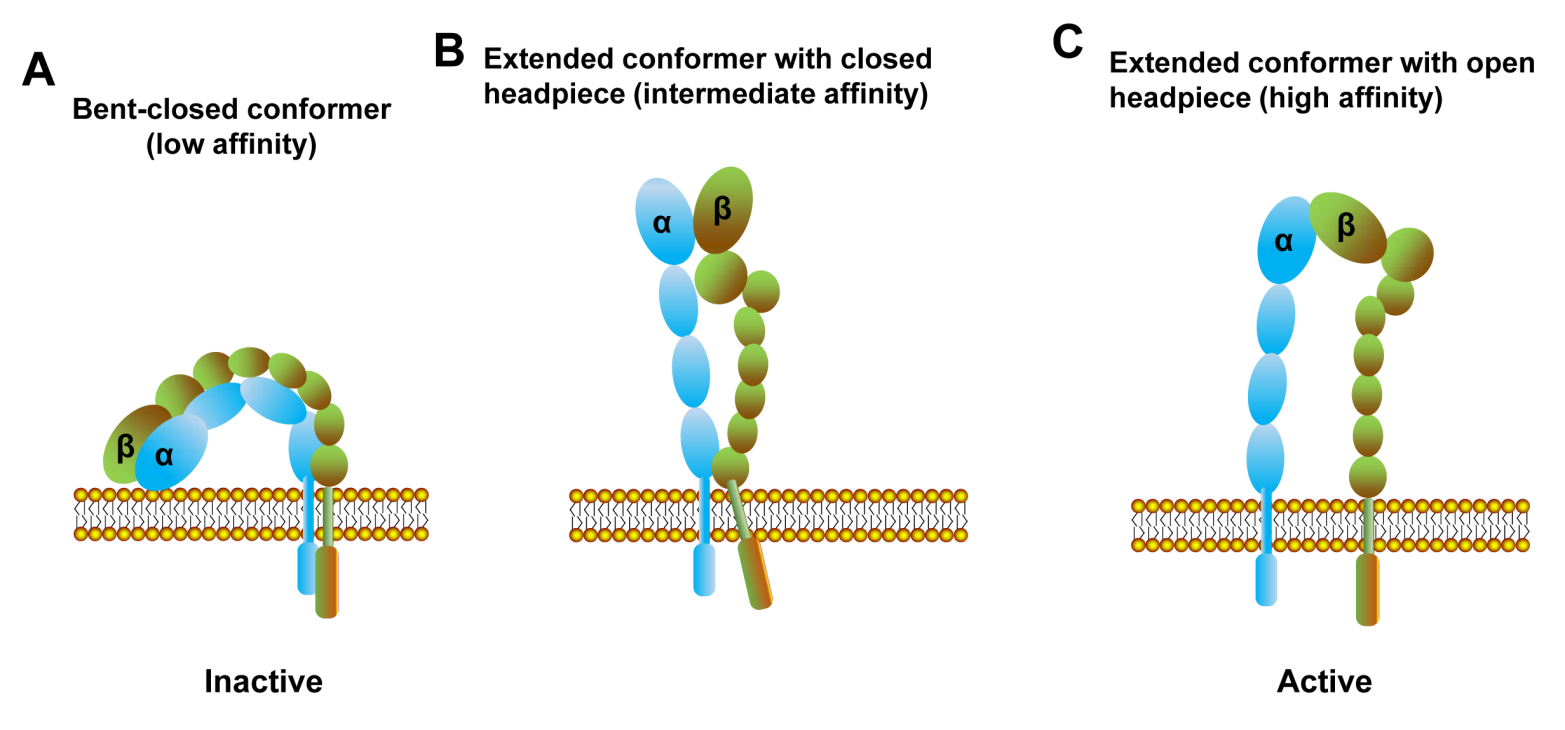
**Representative conformations of integrins**. (A) Bent closed conformer (low affinity). (B) Extended conformer with closed headpiece (intermediate affinity). (C) Extended conformer with open headpiece (high affinity).

Integrins are heterodimeric transmembrane glycoprotein receptors, each comprising an α subunit and a β subunit. To date, 18 α and 8 β subunits have been identified, forming at least 24 functionally distinct integrin heterodimers through non-covalent interactions in mammals [18]. Both α and β subunits are type Ⅰ transmembrane proteins with a large extracellular domain, a single transmembrane helix, and a short unstructured cytoplasmic tail (except β4 subunit) (Fig. 1). ECM ligand-binding sites reside either in the inserted (Ⅰ) domain of the α subunit (in integrins with an α Ⅰ domain, including α1, α2, α10, α11, αD, αX, αM, and αL) or in a pocket epitope formed by the α and β subunits (in α Ⅰ domain-less integrins) (reviewed in [25]). There are three divergent integrin conformations, including bent-closed conformer (low affinity), extended conformer with closed headpiece (intermediate affinity), and extended conformer with open headpiece (high affinity) (Fig. 1). In resting cells, integrins adopt in the inactive bent-closed conformation. Upon binding to ligands, integrins are activated to form the high-affinity conformation [25]. Integrins are able to bind to diverse ligands, and many ECM ligands and membrane-spanning molecules can engage distinct integrin heterodimers [18].

### 2.2 Classification of Integrins

Traditionally, according to their extracellular ligands, integrins can be classified into four subsets [25]: (1) arginine (Arg)-glycine (Gly)-aspartate (Asp) (RGD)-binding integrins (e.g., αvβ1, αvβ3, αvβ5, αvβ6, αvβ8, α5β1, α8β1, and αIIbβ3), which recognize the RGD tripeptide motif in ECM proteins, such as fibronectin, tenascin, vitronectin, fibrinogen, and fibrillin; (2) leukocyte cell-adhesion integrins (e.g., α4β1, α9β1, αLβ2, αMβ2, αXβ2, αDβ2, α4β7, and αEβ7), which mainly regulate inflammation and—most especially the β2-containing integrins—are constitutively expressed in leukocytes; (3) collagen (GFOGER)-binding integrins (e.g., α1β1, α2β1, α10β1, and α11β1), which bind GFOGER-like sequences in collagen; (4) laminin-binding integrins (e.g., α3β1, α6β1, α6β4, and α7β1), which recognize the C-terminal domain of laminins.

### 2.3 Integrin Signaling

Unlike other receptors, integrins transmit biological signals bidirectionally between the extracellular environment and intracellular pathways, encompassing both ‘inside-out’ and ‘outside-in’ mechanisms.

#### 2.3.1 Inside-out Signals

The bent-closed conformation of integrin is usually maintained by endogenous inhibitory proteins, which regulate integrin activity through interaction with integrin’s cytosolic domain, a process referred to as inside-out signaling. That is, intracellular components such as talins (talin-1 and talin-2), kindlins (kindlin-1, kindlin-2, and kindlin-3) bind to the β subunit cytoplasmic tail, thereby inducing dissociation of the α and β cytoplasmic domains and triggering a high-affinity extended conformation that increases the sensitivity of integrins to extracellular ligands. Subsequently, integrins may cluster into diverse adhesive complexes [18,25].

#### 2.3.2 Outside-in Signals

The outside-in signaling is a process whereby extracellular components bind to integrins and trigger downstream signaling cascades. After binding to ECM ligands, integrins cluster and induce the recruitment of adaptor and signaling proteins, including focal adhesion kinases (FAK), SRC proto-oncogene, non-receptor tyrosine kinase (Src), which further activate downstream signaling pathways, such as extracellular signal-regulated kinases (ERK) 1/2, nuclear factor-kappa B (NF-κB), phosphatidylinositol 3-kinase (PI3K)/protein kinase B (Akt), Ras homologous gene family (Rho)/Rho-associated coiled-coil-containing protein kinase (ROCK), Yes-associated protein (YAP)/transcriptional co-activator with PDZ-binding motif (TAZ), and canonical transforming growth factor-beta (TGF-β)/Sma- and Mad-related protein (Smad) [26,27,28,29,30,31,32,33]. These pathways are indispensable for many integrin-dependent cellular processes, including cell survival, adhesion, differentiation, motility, and ECM assembly and remodeling [18,25].

## 3. The Functions and Mechanisms of Integrin in AA and AD

Integrins are critically involved in the development of AA and AD. To date, many rodent animal models have been developed in laboratories to elucidate mechanisms and treatment strategies of AAD (reviewed in [34,35]). However, none perfectly mirrors human disease; each replicates only selected pathological features. Here, we review and discuss the functions and mechanisms of integrins in AAD across distinct rodent and *in vitro* models (Table 1, Ref. [22,23,24,33,36,37,38,39,40,41,42,43,44,45,46,47,48,49,50,51,52,53,54,55,56,57]).

**Table 1.** **The functions and mechanisms of integrins in AAD**.

Integrin subfamily	AAD type	Expression changes	Functions	Mechanism	Experimental model	Ref
αvβ3	AAA	Up	Protective	Activate PI3K/Akt axis and inhibit matrix degradation in VSMCs and macrophages, activate TGF-β/Smad signaling and upregulate contractile genes in VSMCs; inhibit M1 macrophage polarization	Human, Ang II-infused *ApoE^-/-^* mice, cell culture	[33,36,37,38]
	MFS-related TAA	Up	Promoting	Increase proliferative and migratory capacities and alter metabolic phenotype of VSMCs	*Fbn1^C1041G/+^* mice, Cell culture	[39]
	Sporadic TAA/TAD	Down	Protective	Promote Rho GTPase activation and the transcription of VSMC differentiation markers, promote the internalization and degradation of apoptotic VSMCs by macrophages	BAPN-induced mice, cell culture	[24,40,41]
α5β1	AAA	Down	NR	NR	Human	[42]
	*ACTA2*-related heritable TAA/TAD	Down	Protective	Promote VSMC contractile phenotype	VSMCs isolated from *Acta2^R149C/+^* mice	[43]
	MFS-related TAA/TAD	NR	Promoting	Increase inflammation and decrease VSMC contractile phenotype	*Fbn1^mgR/mgR^* mice	[23]
	Acute type A AD	Down	Protective	Suppress apoptosis and elevate proliferation of VSMCs	Human, cell culture	[44,45]
α4β1	AAA	Up	Promoting	Activate FAK/Src pathway, induce T cell migration and adhesion, increase the adhesion of monocytes/macrophages to activated endothelial cells	Elastase-induced and HHcy-aggravated mice, cell culture	[46,47]
α9β1	AD	Down	Protective	Induce contractile phenotypes of VSMCs	Human; cell culture	[48]
αMβ2	AAA	Up	Promoting	Induce macrophage adhesion, reduce macrophage M2 polarization; promote neutrophil recruitment	Human, CaCl_2_‐induced mice, elastase- and CaPO4-induced mice	[49,50]
	TAD	Up	Promoting	Induce adhesion of macrophages and neutrophils	Human, BAPN and Ang II-induced mice, cell culture	[51,52]
α2β1	Sporadic TAD	Up	NR	NR	Human	[53,54]
	Non-syndromic familial TAA/TAD	Down	Protective	Maintain cell adhesion and VSMC contractility	Mice with *Myh11* lysine K1256 deletion, VSMCs isolated from *Acta2^R149C/+^* variant mice	[43,55]
α7β1	AAA	Down	NR	NR	CaCl_2_-induced mice	[56]
	TAA	NR	Protective	Inhibit TGF-β1 maturation and secretion, followed by inactivate the Smad2/3 and ERK1/2 pathways in VSMCs	Transverse aortic constriction-induced mice, *Fbn1^C1041G/+^* mice, BAPN-treated mice	[57]
α6	AAA	Up	Promoting	Promote the phenotypic switch to synthetic phenotype through activating the FAK/STAT3 pathway	Human, Ang II-induced mice, cell culture	[22]
α3β1	AD	Down	NR	NR	Human	[44]

AAD, aortic aneurysm and dissection; NR, not reported; AAA, abdominal aortic aneurysm; TAA, thoracic aortic aneurysm; MFS, Marfan syndrome; AD, aortic dissection; TAD, thoracic aortic dissection; Ang II, angiotensin II; BAPN, β-Aminopropionitrile; PI3K, phosphatidylinositol 3-kinase; TGF-β, transforming growth factor-beta; Smad, Sma- and Mad-related protein; FAK, focal adhesion kinases; Src, SRC proto-oncogene, non-receptor tyrosine kinase.

### 3.1 RGD-Binding Integrins

#### 3.1.1 Integrin αvβ3

β3 integrin (encoded by *ITGB3*) partners with αv integrin to form the αvβ3 integrin, which plays critical roles in the proliferation, migration, and phenotypic differentiation of VSMCs [58]. Integrin αvβ3 is highly expressed in vascular tissues [59], and its expression is markedly altered after injury [60,61,62]. Accumulating evidence links αvβ3 integrin signaling to the development of AA and AD.

Single-cell RNA sequencing data of human AAA and angiotensin Ⅱ (Ang Ⅱ)-infused apolipoprotein E-knockout (*ApoE^-/-^*) mouse AAA models reveal an increase in pro-inflammatory M1-like macrophages and a decrease in VSMC contractile function. Notably, integrin αvβ3 exhibits high expression levels on the surfaces of both macrophages and VSMCs in AAA lesion sites [36]. Integrin αvβ3 seems to exert protective effects in AAA, and several molecules and drugs have been shown to act in AAA by regulating or interacting with αvβ3. For example, in Ang Ⅱ-treated *ApoE^-/-^* mice, omentin attenuated AAA formation via PI3K/Akt activation in an αvβ3-dependent manner in macrophages and VSMCs [37]. An αvβ3-neutralizing antibody abrogated omentin-induced inhibition of matrix metalloproteinase 9 (MMP9) and MMP2 expression in macrophages and VSMCs, respectively [37]. Elevated levels of solute carrier family 44 member 2 (SLC44A2) in the aortas of human AAA and Ang Ⅱ-infused *ApoE^-/-^* AAA mice preserve the VSMC contractile phenotype through interactions with NRP1 and ITGB3, thereby triggering TGF-β/Smad signaling activation and upregulating contractile genes [33,38]. Additionally, a biocompatible nanodrug, EVMS@R-HNC, has been demonstrated to ameliorate Ang Ⅱ-induced AAA progression in mice through specifically binding to integrin αvβ3. This nanodrug comprises the multifunctional drug everolimus (EVMS) encapsulated within hepatitis B virus core protein engineered to present the RGD motif. The therapeutic effects of EVMS@R-HNC are achieved through two main mechanisms: (1) targeting macrophages to inhibit M1 macrophage polarization, thereby leading to a rebalance of the inflammatory microenvironment, and (2) targeting VSMCs to preserve their normal contractile functions [36].

Parker et al. [39] employed *Fbn1^C1041G/+^* mice that replicate a rarely described form of MFS characterized by late-onset, slow-developing TAA that seldom progresses to acute AD or rupture [63], and is accompanied by age-dependent changes in aortic root diameter and wall stiffness [64]. Using this model, they demonstrated that the expression levels of αvβ3 integrin and its ligand vitronectin were both upregulated in the aorta of aged MFS mice compared with wild-type mice. They claimed that β3 integrin overexpression and activation with vitronectin accelerated TGF-β-induced rapamycin-independent component of mammalian target of rapamycin (Rictor) activation in mouse aortic VSMCs, without affecting canonical TGF-β/Smad signaling. This non-canonical crosstalk resulted in increased proliferative and migratory capacities and an altered metabolic phenotype, changes that mirrored the TAA phenotype observed in MFS* in vivo* [39]. It seems that β3 integrin overexpression and activation might promote TAA in MFS, which is contrary to the role of αvβ3 integrin in AAA as described above. Regrettably, Parker et al. [39] did not investigate the phenotype switch of VSMCs *in vitro*. Additionally, they did not explore the role of β3 integrin in MFS-related TAA *in vivo*. Hence, further research is needed to clarify the effect of integrin αvβ3 in this condition.

**Fig. 2.**
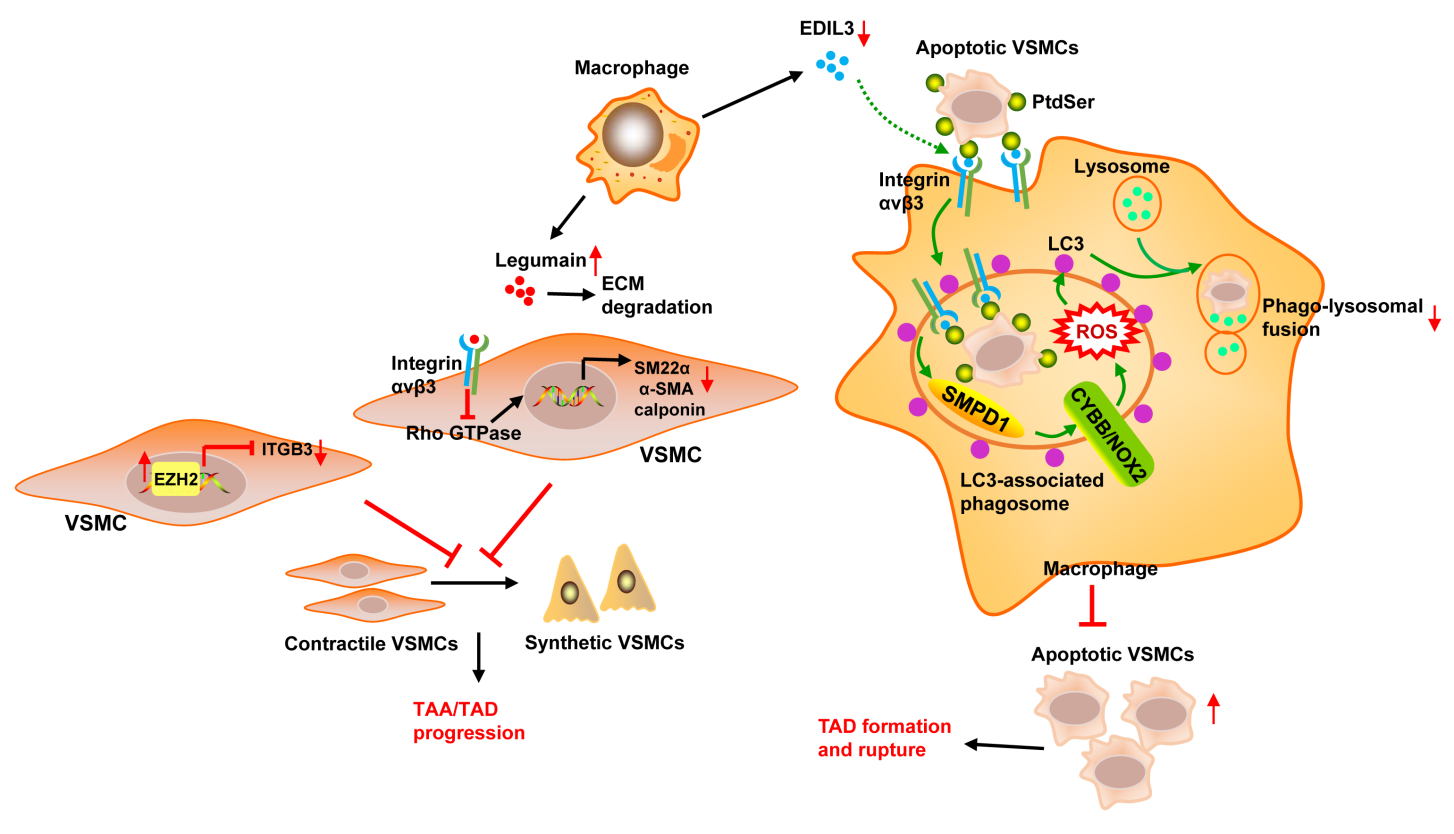
**The mechanisms of integrin αvβ3 in sporadic thoracic AA (TAA) and thoracic AD (TAD)**. Enhancer of zeste homolog 2 (EZH2) facilitates the contractile-to-synthetic phenotype transition in mouse primary vascular smooth muscle cells (VSMCs) by suppressing *ITGB3* expression through binding to the *ITGB3* promoter. Macrophage-derived legumain not only directly induces extracellular matrix (ECM) degradation but also blocks VSMC integrin αvβ3 by direct binding, leading to Ras homologous gene family (Rho) GTPase inactivation. This downregulates the transcription of VSMC differentiation markers, including smooth muscle protein 22 alpha (SM22α), α-smooth muscle actin (α-SMA), and calponin, leading to the suppression of VSMC transformation into a synthetic phenotype. Additionally, macrophage-derived epidermal growth factor-like repeats and discoidin I-like domains 3 (EDIL3) act as a bridge molecule to promote the internalization phase (phagocytosis) through interacting with phosphatidylserine (PtdSer) on apoptotic VSMCs and binding to the macrophage αvβ3 integrin. Following engulfment, EDIL3 enhances sphingomyelin phosphodiesterase 1 (SMPD1) activity in the phagosome by blocking αvβ3 integrin, which facilitates phagosomal reactive oxygen species (ROS) production by NADPH oxidase cytochrome b-245, beta polypeptide (CYBB/NOX2), leading to the promotion of phago-lysosomal fusion. Red arrows: ↑ indicates increase, ↓ indicates decrease.

β-Aminopropionitrile (BAPN) administration in mice has been found to disrupt the cross-linking of collagen and elastin, leading to increased mechanical stress and the development of TAA and thoracic AD (TAD), making this model suitable for studying mechanical stress-induced sporadic TAA and TAD [65,66,67]. *ITGB3* expression is downregulated in the aortic media of TAD patients and BAPN-treated mice [40]. Integrin αvβ3 has been shown to protect against sporadic TAA/TAD, and its expression and activity can be modulated by several molecules. For instance, enhancer of zeste homolog 2 (EZH2), whose expression is elevated in the aortic media of TAD patients and BAPN-induced TAA/TAD mice, facilitates the contractile-to-synthetic phenotype transition in mouse primary VSMCs by suppressing *ITGB3* expression through binding to the *ITGB3* promoter [40] (Fig. 2). Legumain is upregulated in the aorta and serum of TAD patients and in BAPN-induced TAD mice; within the aortic wall, it is primarily localized to macrophages. Macrophage-derived legumain not only directly induces ECM degradation but also blocks VSMC integrin αvβ3 by direct binding, leading to Rho GTPase inactivation. This downregulates the transcription of VSMC differentiation markers, including smooth muscle protein 22 alpha (SM22α), α-smooth muscle actin (α-SMA), and calponin, and exacerbates vascular degeneration, leading to the acceleration of BAPN-induced TAD progression in mice [24] (Fig. 2). Levels of epidermal growth factor-like repeats and discoidin I-like domains 3 (EDIL3) are decreased in the serum and aortic tissues of BAPN-induced TAD mice, accompanied by the accumulation of apoptotic VSMCs in the damaged aorta, which increases the risk of TAD formation and rupture. *Edil3* deficiency causes inefficient internalization and degradation of apoptotic VSMCs. Mechanically, macrophage-derived EDIL3 acts as a bridge molecule to promote the internalization phase (phagocytosis) through interacting with phosphatidylserine (PtdSer) on apoptotic VSMCs and binding to the macrophage αvβ3 integrin. Following engulfment, EDIL3 enhances sphingomyelin phosphodiesterase 1 (SMPD1) activity in the phagosome by blocking αvβ3 integrin, which facilitates phagosomal reactive oxygen species (ROS) production by NADPH oxidase cytochrome b-245, beta polypeptide (CYBB/NOX2), leading to the promotion of phago-lysosomal fusion [41] (Fig. 2).

#### 3.1.2 Integrin α5β1

Integrin α5β1 is a heterodimer composed of the α5 (encoded by *ITGA5*) and β1 subunits. It is constitutively expressed in normal aortas, exclusively localized to the media in humans [42] and to the intima and media in mice [68], and is crucial for the regulation of vessel wall contractility [43]. Expression of α5β1 integrin is decreased in the aortic specimens of AA and AD, and this reduction is associated with disease development [42,43,44,45].

The mRNA expression of integrin α5 and β1 subunits was lower in human AAA specimens than that in healthy aortic tissues. Immunohistochemical staining confirmed decreased α5β1 immunoreactivity in the degraded media of human AAA specimens, accompanied by reduced VSMC density, indicating the coexistence of medial degeneration and α5β1 integrin loss in AAA tissues [42]. To date, the role of integrin α5β1 in AAA has not been reported. Mutations in the *ACTA2* gene, which encodes α-SMA, can impair vascular smooth muscle function and increase the risk of heritable TAA and TAD [43]. In aortic VSMCs isolated from *Acta2^R149C/+^* variant mice, the recruitment of α5β1 integrin at cell-matrix adhesions was decreased, which reduced VSMC contractility [43]. This cell model suggests that integrin α5β1 might exert a protective effect on *ACTA2*-related heritable TAA/TAD. However, using an α5/2 chimera (in which the α5 tail was replaced with that of integrin α2), Chen et al. [23] demonstrated that fibronectin accumulation in the tunica media promoted TAA progression through binding to the cytoplasmic domain of integrin α5, leading to increased inflammation and a decrease in VSMC contractile phenotype in *Fbn1^mgR/mgR^* MFS mice. These mice recapitulate the most commonly diagnosed form of MFS, characterized by early-onset, progressive TAA that invariably culminates in TAD and rupture [63]. Thus, integrin α5β1 appears to exert a promoting effect of MFS-related TAA/TAD.

Through proteomic analysis and immunohistochemistry, Xing et al. [44] demonstrated that integrin α5 was significantly downregulated in aortic tissues of acute type A AD patients compared with healthy donors. This downregulation is mainly in the cytomembrane and cytoplasm. Consistently, Xue and his co-workers found that compared with the corresponding controls who died from traffic accident, levels of integrin α5β1 were dramatically reduced in patients with acute type A AD, in whom FAK content was also decreased while caspase-3 levels were enhanced [45]. Downregulation of integrin α5β1 expression using siRNA technology significantly elevated apoptosis and suppressed proliferation of HASMCs. These results indicate that the integrin α5β1-FAK signaling pathway may exert a protective role during the onset of acute type A AD [45]. However, the exact role and mechanism of integrin α5β1 in AD require further experimental investigation.

### 3.2 Leukocyte Cell-Adhesion Integrins

#### 3.2.1 Integrin α4 (α4β1/VLA-4)

Integrin α4 and β1 subunits are constitutively expressed in normal aortas [42]. Miao et al. [46] have demonstrated that autotaxin, which aggravates AAA development, upregulates the mRNA and protein levels of integrin α4 in T cells. Moreover, autotaxin triggers the migration and adhesion of T cells through binding to integrin α4 and activating the FAK/Src pathway. Ren et al. [47] have shown that andrographolide ameliorates AAA progression partially by suppressing integrin α4 expression in monocytes/macrophages and decreasing the adhesion of these cells to activated endothelial cells. These results indicate that integrin α4-dependent cell activation may critically promote inflammatory leukocyte recruitment and thereby drive AAA progression.

#### 3.2.2 Integrin α9β1/α9/ITGA9

Integrin α9 (encoded by *ITGA9*) specifically binds to β1 subunit to form the α9β1 heterodimer, which is known to stabilize the adhesion of leukocytes to activated endothelium [69]. Huang et al. [48] found that integrin α9 was significantly decreased in the ascending aortic tissues of patients with acute AD using both whole-genome transcriptional microarray and western blot analysis. Downregulation of *ITGA9* in aortic VSMCs resulted in the switch to a synthetic phenotype, whereas its overexpression promoted the switch to a contractile phenotype [48]. This cell model indicates that integrin α9β1 may inhibit AD progression; however, further animal models are needed to confirm this finding.

#### 3.2.3 Integrin αMβ2/αM (ITGAM)

Integrin αM (also known as CD11b) is encoded by the integrin subunit alpha M (*ITGAM*) gene. It is not only a surface biomarker of myeloid cells, such as monocytes, macrophages, dendritic cells, but also an essential adhesion molecule. Integrin αM can only form a heterodimer with β2 integrin (encoded by *ITGB2*), resulting in the formation of the αMβ2 complex, also known as macrophage-1 antigen (Mac-1).

**Fig. 3.**
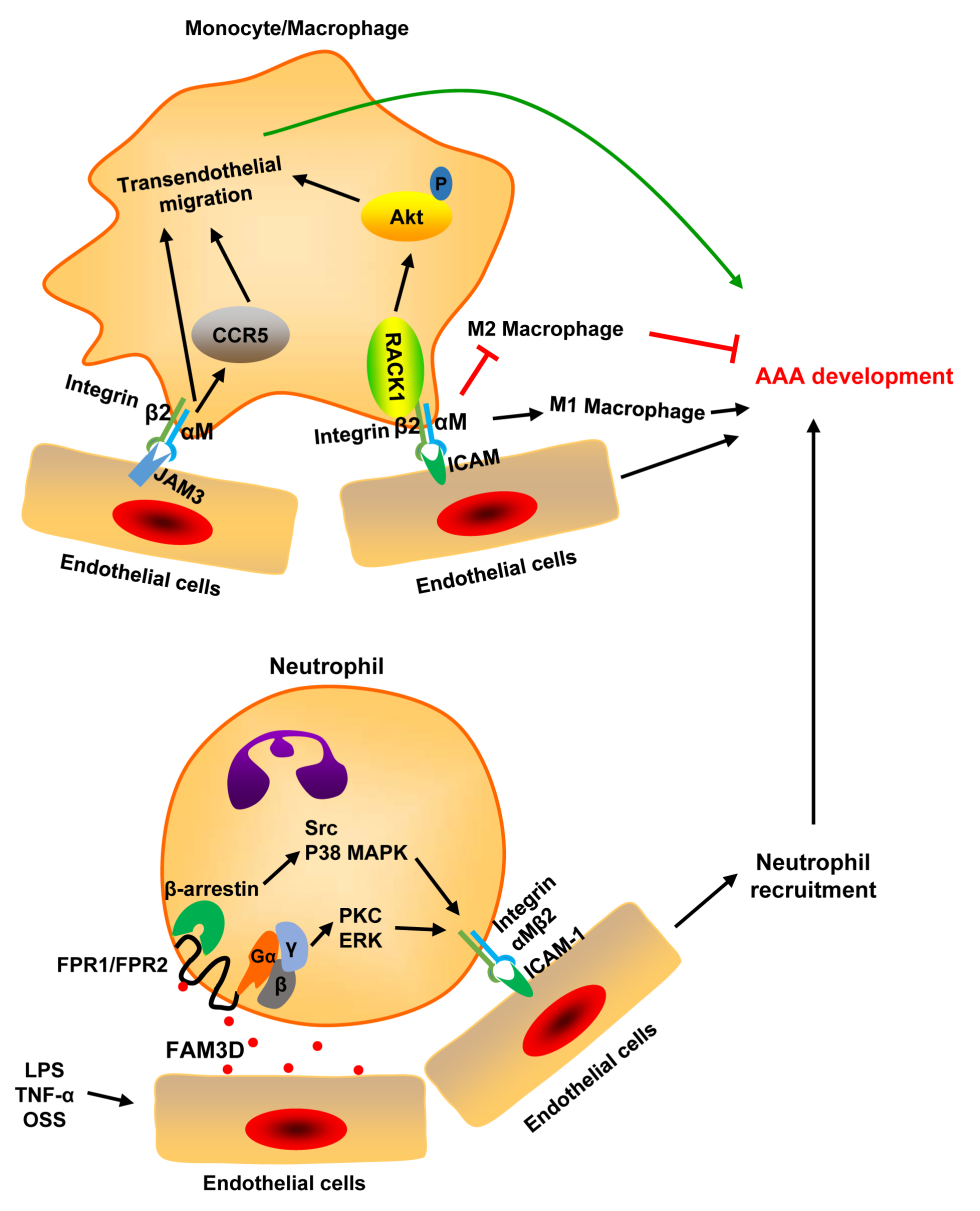
**The functions and mechanisms of integrins αMβ2 in abdominal AA (AAA)**. (1) Monocytes/macrophages‐derived integrin αM specifically recognizes endothelial intercellular adhesion molecule (ICAM), enabling their adhesion to endothelial cells. Integrin αM also induces macrophage M1 polarization and inhibits M2 polarization. Moreover, integrin αM promotes macrophage transendothelial migration by increasing C-C chemokine receptor type 5 (CCR5) expression, activating the protein kinase B (Akt) pathway via receptor for activated C kinase 1 (RACK1), and enhancing the association with junctional adhesion molecule 3 (JAM3). These effects contribute to AAA development. (2) Pathological stimuli including lipopolysaccharide (LPS), tumor necrosis factor α (TNF-α), or oscillatory shear stress (OSS) induce the expression and secretion of family with sequence similarity 3, member D (FAM3D) in endothelial cells. The FAM3D activates integrin αMβ2 via formyl peptide receptor (FPR) 1 and FPR2 via Gi protein and β-arrestin signals. Then, the activated integrin αMβ2 binds to endothelial ICAM-1 leading to neutrophil recruitment and AAA development. ERK, extracellular signal-regulated kinases.

Integrin αM was upregulated in human AAA tissues. Moreover, in CaCl_2_‐induced AAA mouse models, integrin αM expression progressively increased with time [49]. Although *Itgam*-knockout (*Itgam^‐/‐^*) mice did not show a reduced incidence of AAA, deletion of *Itgam* alleviated AAA development, decreased vascular structural injury, and suppressed inflammatory responses. Moreover, knockout of *Itgam* reduced peripheral blood macrophage recruitment into the aorta and promoted macrophage M2 polarization. Mechanistically, *Itgam* deficiency not only impaired macrophage adhesion to endothelial cells, but also inhibited macrophage transendothelial migration by reducing C-C chemokine receptor type 5 (CCR5) expression, inhibiting Akt pathway activation via receptor for activated C kinase 1 (RACK1), and decreasing the association with junctional adhesion molecule 3 (JAM3) [49,70] (Fig. 3). The expression and activity of integrin αMβ2 can be regulated by several drugs and molecules. For example, Akimoto et al. [71] revealed that inhibition of NF-κB using a decoy oligodeoxynucleotide restrained AAA development in CaCl_2_-induced rats, accompanied by reduced expression of integrin αM. Endothelial cell-derived family with sequence similarity 3, member D (FAM3D) has been found to promote the activation of neutrophil integrin αMβ2 through formyl peptide receptor (FPR)-related Gi protein and β-arrestin signaling. Activated integrin αMβ2 binds to endothelial intercellular adhesion molecule (ICAM)-1, contributing to neutrophil recruitment and AAA development [50] (Fig. 3). These results indicate that integrin αM/αMβ2 is an attractive therapeutic target in AAA treatment.

Wang et al. [51] found that both integrin αM and β2 were significantly upregulated in the aortic tissues of Type A acute AD patients. Li et al. [52] and co-workers also demonstrated elevated integrin αM and β2 in the aortic samples from TAD patients. Consistently, integrin αMβ2 was upregulated in the ascending aortas of TAD mice treated with BAPN and Ang II [52]. During TAD development, integrin αMβ2 was predominantly colocalized with its ligand fibrinogen, which was upregulated and mainly deposited in aortic adventitia prior to the occurrence of TAD and contributed to disease progression through recruiting macrophages and neutrophils. Inhibition of fibrinogen-integrin αMβ2 interaction using integrin antagonistic polypeptides attenuated fibrinogen-induced adhesion of macrophages and neutrophils. Downregulation of fibrinogen with batroxobin or in heterozygous fibrinogen γ-chain knockout (*Fgg^+/-^*) mice inhibited the infiltration of CD45^+^ and integrin αM^+^ inflammatory cells and reduced TAD incidence [52]. These results indicate that disruption of the fibrinogen-integrin αMβ2 interaction may provide a potential therapeutic strategy against TAD.

### 3.3 Collagen (GFOGER)-Binding Integrins

#### Integrin α2β1/ITGA2

The α2β1 integrin, also known as VLA-2, GPIa-IIa, and CD49b, is an obligate heterodimer composed of the α2 integrin subunit (encoded by *ITGA2*) and the β1 integrin subunit. Analysis of RNA-sequencing data from the GSE52093 and GSE153434 datasets, Lian et al. [53] found that *ITGA2* was significantly upregulated in the ascending aorta of patients with acute type A AD. Qiu and coworkers demonstrated that compared with myocardial infarction patients, ITGA2 was highly expressed in the aortic tissues and serum samples of sporadic TAD patients. Moreover, serum ITGA2 can accurately discriminate TAD from myocardial infarction (AUC = 0.801, 95% CI: 0.691–0.911) [54]. However, the role of ITGA2 in sporadic TAD remains to be explored. Integrin α2β1 is downregulated and seems to exert a protective effect in non-syndromic familial TAA/TAD. For example, *ITGA2* was downregulated in the aorta of mice with myosin heavy chain (*Myh11*) lysine K1256 deletion [55]. This downregulation, which results in suboptimal cell adhesion, might be responsible for defective aortic contraction, thereby increasing hemodynamic stress and contributing to TAD [55]. In aortic VSMCs isolated from *Acta2^R149C/+^* variant mice, the recruitment of α2β1 integrin at cell-matrix adhesions was decreased, reducing VSMC contractility. This reduction in contractility may contribute to the development of TAA [43].

### 3.4 Laminin-Binding Integrins

#### 3.4.1 Integrin α7β1/α7/ITGA7

The integrin α7β1, composed of the α7 (encoded by *ITGA7*) and β1 subunits, is highly expressed in the late fetal heart and adult cardiac tissue [72,73]. By analyzing differentially expressed genes in the GSE109639 dataset, Gan et al. [56] found that *ITGA7* was significantly downregulated in CaCl_2_-induced AAA mice compared with the physiological saline-treated sham group. Moreover, it is also a hub gene in the protein-protein interaction network according to their ranking of topological features. Nevertheless, the role of *ITGA7* in AAA remains unexplored. Studies have demonstrated the association between integrin α7β1 and TAA [57,74]. Integrin α7 serves as the receptor for angiogenic factor with G-patch and FHA domains 1 (AGGF1) on VSMCs through binding to the Arg-Asp-Asp (RDD) motif of AGGF1 [74]. AGGF1 expression was decreased in three distinct murine TAA models, namely transverse aortic constriction, *Fbn1^C1041G/+^*, and BAPN treatment, as well as in human TAA patients. Heterozygous *Aggf1^+/-^* and VSMC-specific *Aggf1 *knockout (*Aggf1^smcKO^*) mice developed exacerbated TAA phenotypes. Recombinant AGGF1 protein attenuated TAA phenotypes in mice, whereas deletion of the RDD residues in AGGF1 abolished these protective effects. Similar to *Aggf1* knockdown, downregulation of *Itga7* reduced latency-associated peptide (LAP)-TGF-β1 expression and increased TGF-β1 maturation and secretion in VSMCs. This research group demonstrated that AGGF1 enhanced integrin α7-LAP-TGF-β1 association, blocked LAP-TGF-β1 cleavage into mature TGF-β1, and inhibited Smad2/3 and ERK1/2 phosphorylation in VSMCs [57]. However, integrin α7 expression was not detected in these TAA models. In general, disruption of integrin α7-AGGF1 interaction appears to drive TAA development.

#### 3.4.2 Integrin α6/ITGA6

Integrin α6 (encoded by* ITGA6*) is highly expressed in aortic samples of AAA patients and Ang II-induced AAA mice. Downregulation of *ITGA6* in VSMCs results in the attenuation of Ang II-induced AAA and prevents the phenotypic switch to a synthetic phenotype through inactivation of the FAK/STAT3 signal pathway [22], indicating a promoting role of integrin α6 in AAA. The transcription of *ITGA6* is regulated by histidine triad nucleotide-binding protein 1 (HINT1). Pathological stimuli induce nucleoporin 98 (Nup98)-mediated nuclear translocation of HINT1, which enhances its interaction with the transcription factor AP-2α, thereby leading to the upregulation of *ITGA6* [22]. Integrin α6 can form heterodimers with both β1 and β4 subunits; however, Zhang et al. [22] did not specify whether the integrin in question was α6β1 or α6β4.

#### 3.4.3 Integrin α3β1/ITGA3

Integrin α3 (encoded by *ITGA3*) specifically binds to the β1 subunit to form the integrin α3β1 complex. Integrin α3 was significantly downregulated in aortic tissues from patients with acute type A or type B AD compared with healthy donors, indicating it may serve as a novel biomarker for acute AD [44]. However, the role of integrin α3 in AD remains largely unknown.

## 4. Conclusions and Perspectives

As heterodimeric transmembrane receptors, integrins regulate cell-matrix and intercellular crosstalk through inside-out and outside-in signaling pathways. Recent studies have demonstrated the critical role of integrins in the pathogenesis of AA and AD. Abnormal serum expression of integrins in AA/AD may serve as non-invasive diagnostic biomarkers. For example, ITGA2 is highly expressed in serum samples of TAD patients, and serum ITGA2 levels have a good diagnostic value in distinguishing TAD from myocardial infarction [54]. However, currently, there are limited studies investigating serum levels of integrins in AA/AD patients. Additional research is necessary to further elucidate this area.

The functions and mechanisms of integrins in AAD are summarized, offering crucial insights into the pathogenesis of AAD and highlighting potential therapeutic targets to prevent disease progression. Current studies have confirmed that targeting specific integrins can prevent the progression of AA/AD. For example, the biocompatible nanodrug EVMS@R-HNC relieves Ang II-induced AAA progression through specifically binding to integrin αvβ3 in macrophages and VSMCs [36]. Andrographolide ameliorates AAA progression through suppressing integrin α4 expression [47]. Inhibition of the fibrinogen-integrin αMβ2 interaction or downregulation of fibrinogen attenuates TAD occurrence [52]. Enhancing the interaction between integrin α7β1 and AGGF1 can inhibit TAA development [57]. However, these studies are currently limited to preclinical experiments, and it is essential to evaluate their efficacy in clinical settings. Notably, the functions and mechanisms of several other integrins remain largely unexplored and future research is needed to elucidate their roles. Targeting integrins holds significant translational potential for the prevention and treatment of AAD.
